# Structure and Ultrastructure of the Endodermal Region of the Alimentary Tract in the Freshwater Shrimp *Neocaridina heteropoda* (Crustacea, Malacostraca)

**DOI:** 10.1371/journal.pone.0126900

**Published:** 2015-05-21

**Authors:** Lidia Sonakowska, Agnieszka Włodarczyk, Izabela Poprawa, Marcin Binkowski, Joanna Śróbka, Karolina Kamińska, Michalina Kszuk-Jendrysik, Łukasz Chajec, Bartłomiej Zajusz, Magdalena Maria Rost-Roszkowska

**Affiliations:** 1 Department of Animal Histology and Embryology, University of Silesia, Katowice, Poland; 2 Department of Biomedical Computer Systems, X-ray Microtomography Lab, University of Silesia, Chorzów, Poland; Fish Vet Group, THAILAND

## Abstract

The freshwater shrimp *Neocaridina heteropoda* (Crustacea, Malacostraca, Decapoda) originates from Asia and is one of the species that is widely available all over the world because it is the most popular shrimp that is bred in aquaria. The structure and the ultrastructure of the midgut have been described using X-ray microtomography, transmission electron microscopy, light and fluorescence microscopes. The endodermal region of the alimentary system in *N*. *heteropoda* consists of an intestine and a hepatopancreas. No differences were observed in the structure and ultrastructure of males and females of the shrimp that were examined. The intestine is a tube-shaped organ and the hepatopancreas is composed of two large diverticles that are divided into the blind-end tubules. Hepatopancreatic tubules have three distinct zones – proximal, medial and distal. Among the epithelial cells of the intestine, two types of cells were distinguished – D and E-cells, while three types of cells were observed in the epithelium of the hepatopancreas – F, B and E-cells. Our studies showed that the regionalization in the activity of cells occurs along the length of the hepatopancreatic tubules. The role and ultrastructure of all types of epithelial cells are discussed, with the special emphasis on the function of the E-cells, which are the midgut regenerative cells. Additionally, we present the first report on the existence of an intercellular junction that is connected with the E-cells of Crustacea.

## Introduction

The tube-shaped digestive system of Crustacea opens anteriorly at the mouthparts and posteriorly at the anus. It is composed of three distinct regions—the fore-, mid- and hindgut. While the fore- and hindgut are lined with the cuticle, the midgut is devoid of any sheets on its surface or may be covered with the peritrophic membrane [1]. The midgut (the endodermal region of the digestive system) in Crustacea may be a long tube that is devoid of any diverticles and caeca [2–4] or it may have anterior caeca [5]. It can be differentiated into the midgut (intestine) and a large lobular gland called the hepatopancreas [6–8] or the midgut may be replaced by a large hepatopancreas [6–8]. The midgut is responsible for the synthesis and secretion of digestive enzymes, absorption, excretion, storage of the reserve material (organic and inorganic reserves), detoxification and nutrient uptake [9,10]. However, the hepatopancreas is treated as the organ that is primarily responsible for digestion, the absorption of nutrients, the accumulation of the reserve material and the excretion and detoxification of xenobiotics [11,12]. Therefore, it is highly sensitive to the physiological and environmental changes that occur in the natural habitats of animals [11].

Numerous types of cells have been described from among the cells of the midgut epithelium (e.g., E—embryonic cells, F—fibrillar cells, etc.). However, there are still many controversies connected with the functions of cells that have been described; some authors seemed to have difficulties in differentiating all of the types of cells. Therefore, in the literature we can still find data that asserts that all of the cells that form the midgut epithelium in Crustacea may represent the stages of cell differentiation [7,10,13–16].

As the material for the study, we used the freshwater shrimp *Neocaridina heteropoda* (Crustacea, Malacostraca, Decapoda), which originates from Asia. However, it is one of the species that is widely available all over the world as it is the most popular shrimp that is bred in aquaria. *N*. *heteropoda* is primarily a phytophagous species that eats mostly algae and plant material. However, it can also feed on the organic matter and microfauna that grows on the leaves of the plants, stones or glass in an aquarium because it is an opportunistic omnivorous species [17]. The aims of our studies were: (a) to present a 3D localization of the midgut inside the body cavity using X-ray microtomography; (b) to describe the precise ultrastructure of the midgut epithelium (intestine and hepatopancreas) in males and females of *N*. *heteropoda*; (c) to determine whether regionalization in the epithelium of hepatopancreatic tubules occurs; (d) to describe the process of the secretion of substances and (e) to determine whether embryonic cells (if they exist) play the role of midgut regenerative cells.

## Results

**Fig 1:**
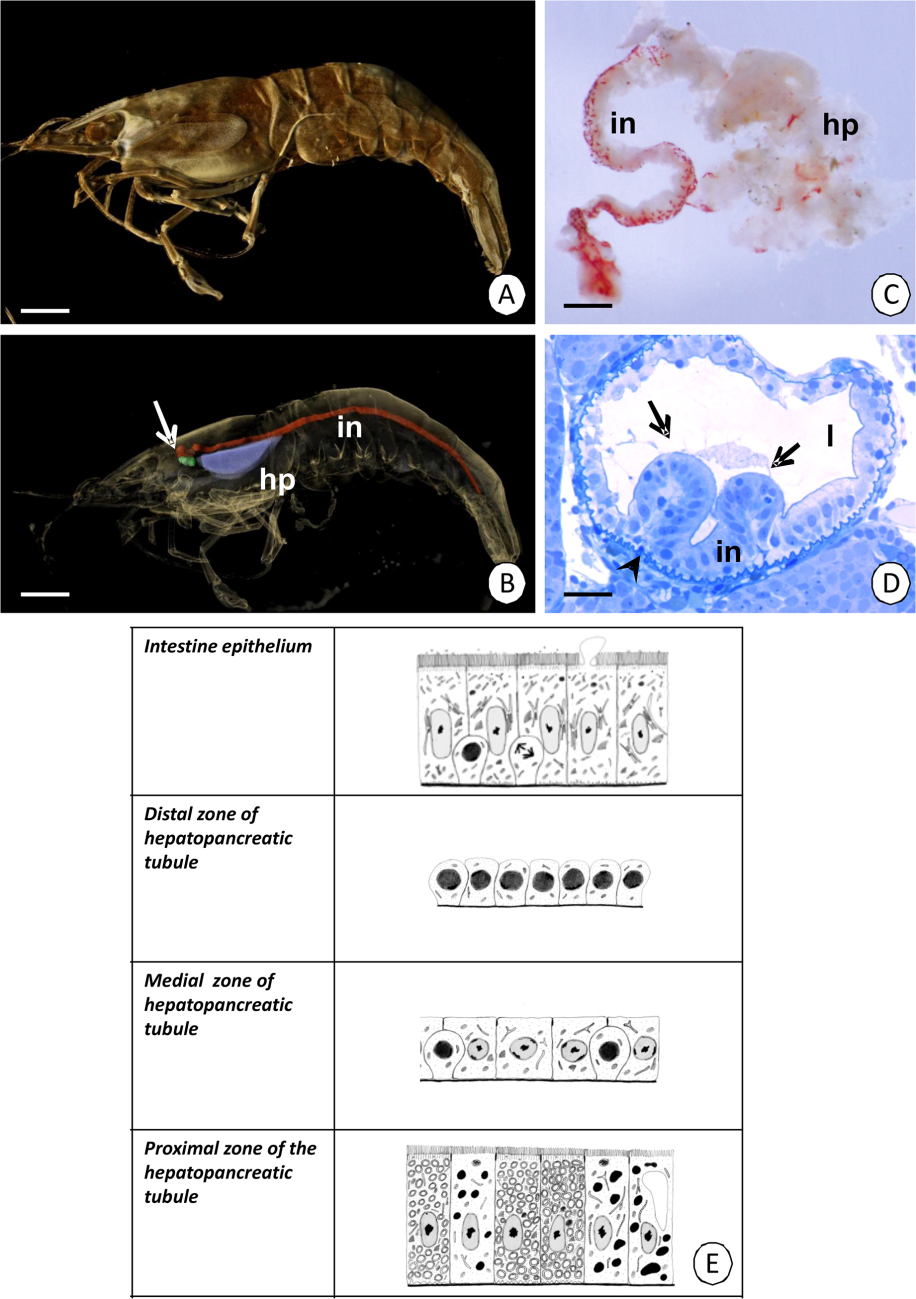
Digestive system of *Neocaridina heteropoda* with the midgut epithelium. (A) *Neocaridina heteropoda*. XMT. Bar = 2 mm. (B-C) A fragment of the digestive system of *N*. *heteropoda*. (B) XMT. Bar = 2 mm. (C) Stereomicroscopy. Bar = 272 µm. (D) The beginning of two diverticles of the hepatopancreas (arrows). Light microscopy. Bar = 28 µm. (E) Diagrammatic representation of epithelia in the intestine and zones of hepatopancreas. Intestine (in), hepatopancreas (hp), stomach (white arrow), midgut lumen (l), intestine epithelium (in), basal lamina (arrowhead).

The midgut of *Neocaridina heteropoda* (Fig 1A), which is located on the dorsal side of the body (S1 Video), is composed of two distinct parts—an intestine that is tubular in shape and a large lobular hepatopancreas (Fig 1B and 1C). The following description relates to both males and females as no sexual differences were observed.

### The structure and ultrastructure of intestine

**Fig 2:**
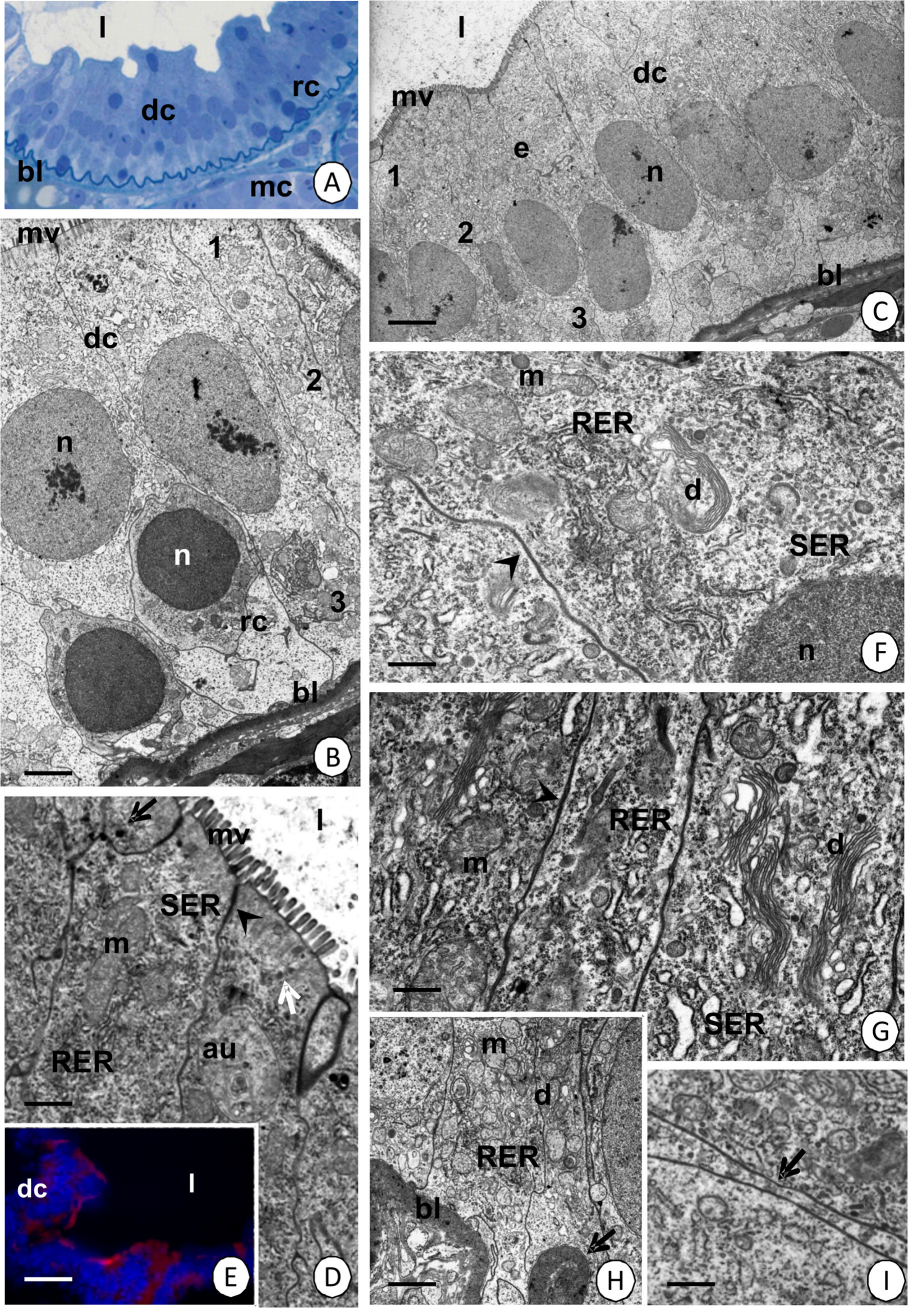
The epithelium of the intestine. (A) Two types of cells—digestive (dc) and regenerative cells (rc). Light microscopy. Bar = 21 µm. (B) Anterior fragment of the intestine with regenerative cells (rc) distributed among the basal regions of the digestive cells (dc). TEM. Bar = 2 µm. (C) The intestine epithelium (e) composed of only digestive cells (dc). TEM. Bar = 3 µm. (D) The apical cytoplasm of the digestive cells. TEM. Bar = 0.87 µm. (E) The apical cytoplasm in the digestive cells (dc) with actin filaments stained in red. Nuclei stained in blue. Fluorescent microscopy. Bar = 24.5 µm. (F-G) The perinuclear region of the cytoplasm. (F) TEM. Bar = 0.65 µm. (G) TEM. Bar = 0.75 µm. (H) Basal cytoplasm of the digestive cells with distinct folds of the basal membrane (arrows). TEM. Bar = 1.46 µm. (I) Gap junction (arrow). TEM. Bar = 0.93 µm. Intestine lumen (l), basal lamina (bl), visceral muscles (mc), nucleus (n), microvilli (mv), regions of the cytoplasm: 1—apical, 2—perinuclear, 3—basal, cisterns of the rough (RER) and smooth (SER) endoplasmic reticulum, autophagosomes (au), electron-dense vesicles (black arrows), electron-lucent vesicles (white arrows), smooth septate junction (arrowheads), mitochondria (m), Golgi complexes (d), septate junction (arrowheads).

The intestine of *N*. *heteropoda* consists of a simple columnar epithelium that lies on the non-cellular basal lamina (Fig 1D). It is separated from the body cavity by the visceral muscles, which form two layers—an inner layer of circular muscles and an outer layer of longitudinal muscles. The epithelium of intestine is composed of two types of cells—digestive and regenerative cells (Figs 1E and 2A). However, the regenerative cells are only distributed in the anterior part of the intestine (Fig 2A and 2B), while the remaining epithelium is composed of only digestive cells (Fig 2C).

#### Digestive cells

The cytoplasm of the columnar digestive cells shows distinct regionalization in the distribution of organelles—basal, perinuclear and apical regions can be distinguished (Fig 2B and 2C). The apical membrane forms long microvilli (Fig 2C and 2D) whose roots, which are made of filaments, enter the apical cytoplasm and form a distinct cortical layer. The cortical layer is poor in organelles (Fig 2E). The apical cytoplasm just below the cortical layer has numerous cisterns of the rough and smooth endoplasmic reticulum, mitochondria, some multivesicular bodies, autophagosomes and vesicles that have an electron-lucent and electron-dense content (Fig 2D). The oval-shaped nucleus contains the euchromatin, which has an electron-medium density (Fig 2B and 2C). The perinuclear region is rich in cisterns of the rough and smooth endoplasmic reticulum, Golgi complexes, but is poor in mitochondria (Fig 2F and 2G). The basal membrane forms small folds. Many mitochondria, cisterns of the rough endoplasmic reticulum and some Golgi complexes accumulate in their neighborhood (Fig 2H).

**Fig 3:**
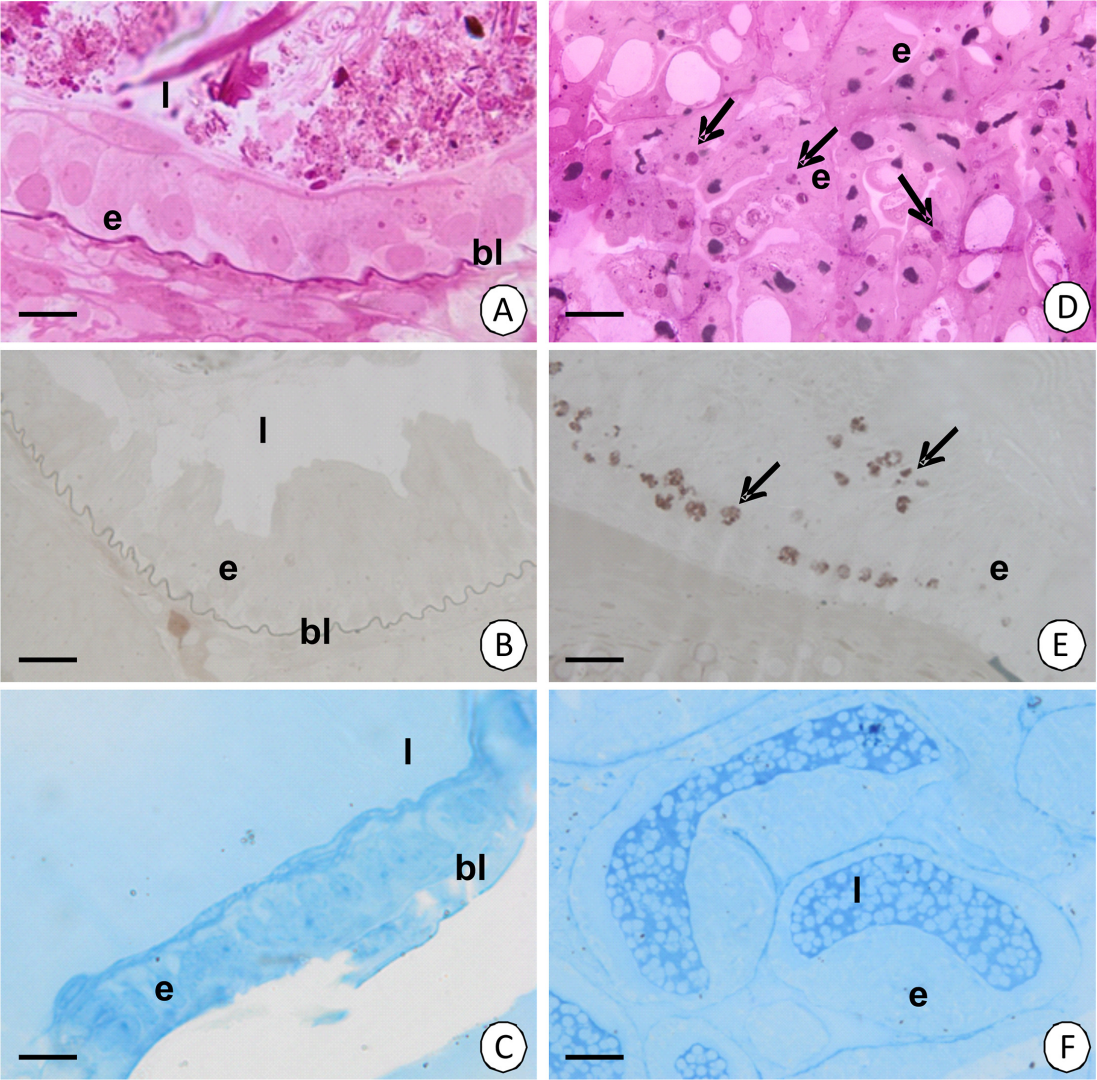
Histochemical staining of the midgut epithelium of *N*. *heteropoda*. (A) Intestine. PAS. Light microscopy. Bar = 10 µm. (B) Intestine. Sudan Black B. Light microscopy. Bar = 10 µm. (C) Intestine. BPB. Light microscopy. Bar = 12 µm. (D) Hepatopancreas. PAS. Light microscopy. Bar = 19 µm. (E) Hepatopancreas. Sudan Black B. Light microscopy. Bar = 12 µm. (F) Hepatopancreas. BPB. Light microscopy. Bar = 21 µm. Midgut epithelium (e), midgut lumen (l), basal lamina (bl), positive reaction (arrows).

The reserve material does not accumulate in the cytoplasm of the digestive cells. Therefore, it is PAS-negative, Sudan Black B-negative and BPB-negative (Fig 3A–3C). Smooth septate junctions can be observed between the cell membranes of the apical regions (Figs 2C and 2D, 6C), while septate junctions (Fig 2F and 2G) and gap junctions (Fig 2I) occur between the membranes of the perinuclear and basal membranes of the adjacent digestive cells.

#### Regenerative cells

**Fig 4:**
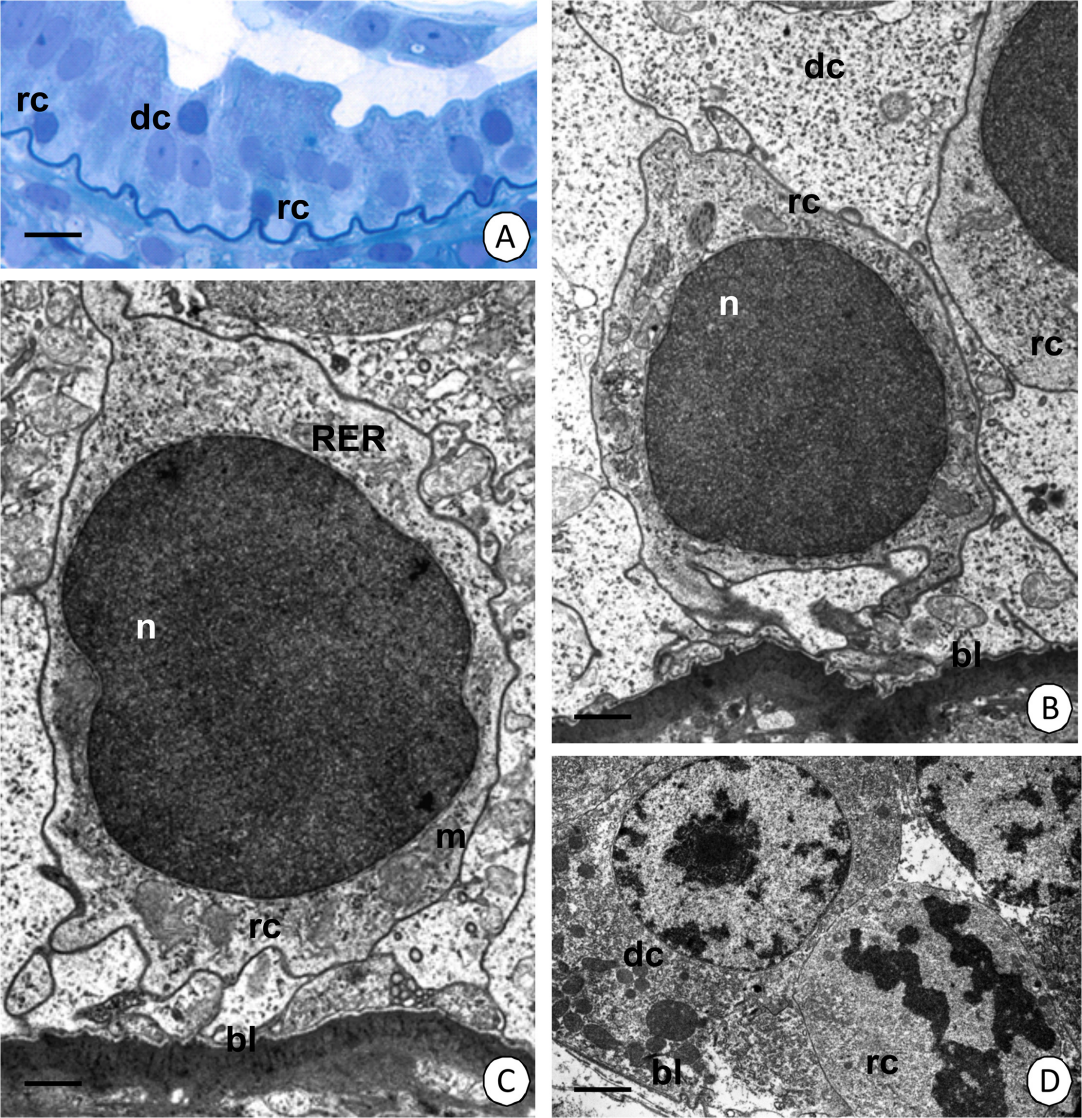
Regenerative cells in the intestine of *N*. *heteropoda*. (A-C) Regenerative cells (rc) situated between the basal regions of the digestive cells (dc). (A) Light microscopy. Bar = 12 µm. (B) TEM. Bar = 0.76 µm. (C) The cytoplasm of the regenerative cells (rc) poor in organelles. TEM. Bar = 0.6 µm. (D) Dividing regenerative cell (rc), digestive cell (dc). TEM. Bar = 3 µm. Nucleus (n), basal lamina (bl), mitochondria (m), cisterns of the rough endoplasmic reticulum (RER).

**Fig 5:**
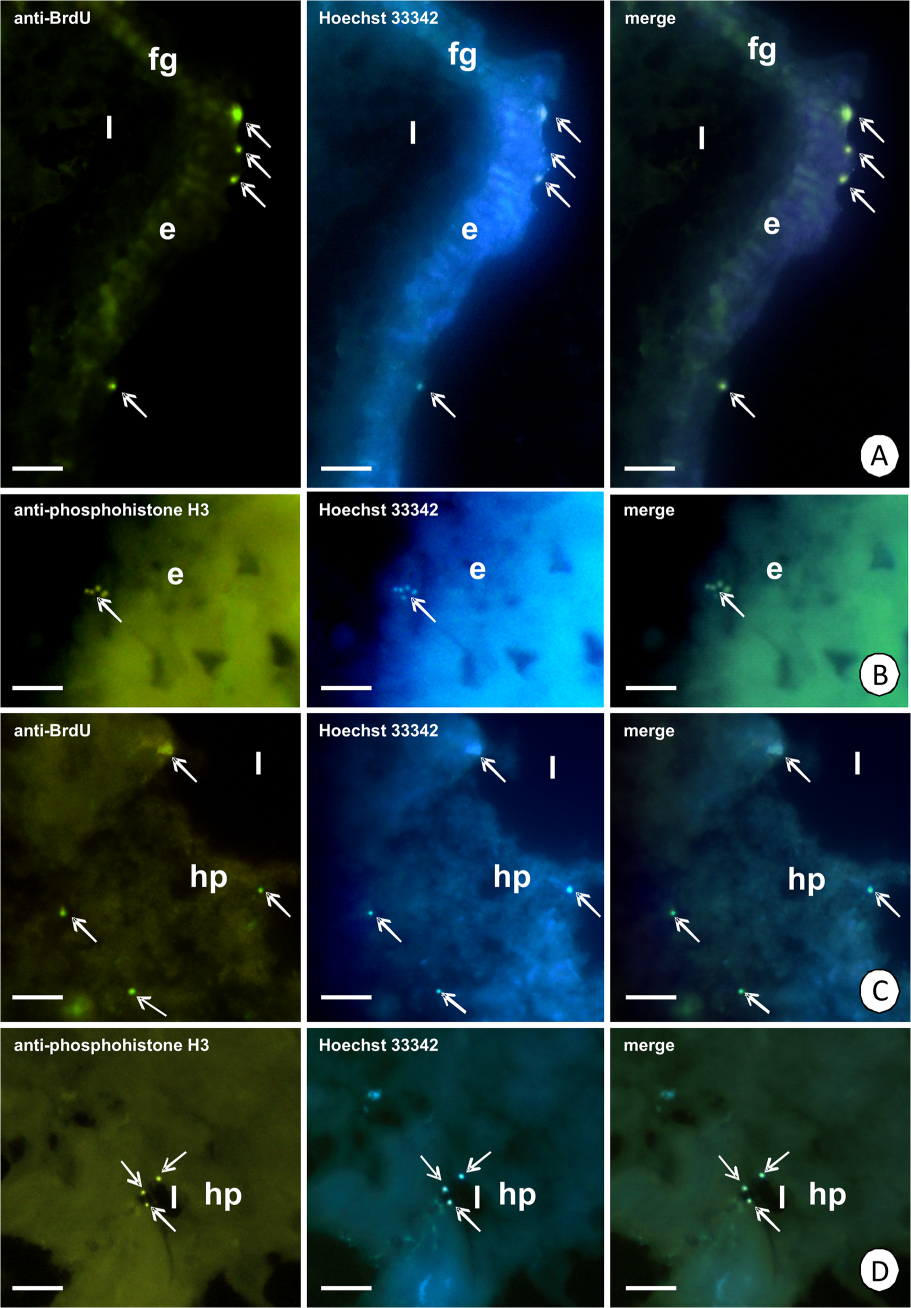
Immunohistochemical detection of cells proliferation. (A) Fluorescence micrograph showing BrdU-labeled regenerative cells (green) (arrows). Longitudinal section of the intestine epithelium (e). Hoechst 33342 staining (blue). Fluorescent microscopy. Bar = 24 µm. (B) Fluorescence micrograph showing anti-phosphohistone H3 (green) regenerative cells (arrows) during mitotic division. Longitudinal section. Hoechst 33342 staining (blue). Fluorescent microscopy. Bar = 13 µm. (C) Fluorescence micrograph showing BrdU-labeled regenerative cells (green) (arrows). Transverse section of the hepatopancreas (hp). Hoechst 33342 staining (blue). Fluorescent microscopy. Bar = 20 µm. (D) Fluorescence micrograph showing anti-phosphohistone H3 (green) regenerative cell (arrows). Cross section of the hepatopancreas (hp). Hoechst 33342 staining (blue). Fluorescent microscopy. Bar = 28 µm. Midgut lumen (l), foregut (fg), intestine epithelium (e), hepatopancreatic lumen (l).

Oval-shaped regenerative cells are situated between the basal regions of the digestive cells (Figs 1E, 4A and 4B) and they are distributed only in the anterior part of the intestine (about ¼ of the length of the intestine) (Figs 2A and 2B, 4A), while the remaining region is devoid of regenerative cells (Fig 2C). They do not contact the midgut lumen. The basal membrane does not form any folds and the cytoplasm is poor in organelles during the interphase (Fig 4B and 4C). Some mitochondria and cisterns of the rough endoplasmic reticulum are distributed around the large oval-shaped nucleus, which shows a high electron-density and is devoid of any distinct patches of heterochromatin (Fig 4B and 4C). Septate junctions were observed between the regenerative and digestive cells. The regenerative cells undergo mitosis (Fig 4D), which was confirmed by the anti-BrdU labeling (Fig 5A) and anti-phosphohistone H3 labeling (Fig 5B). The process of the differentiation of the regenerative cells was not observed.

#### Types of secretion

**Fig 6:**
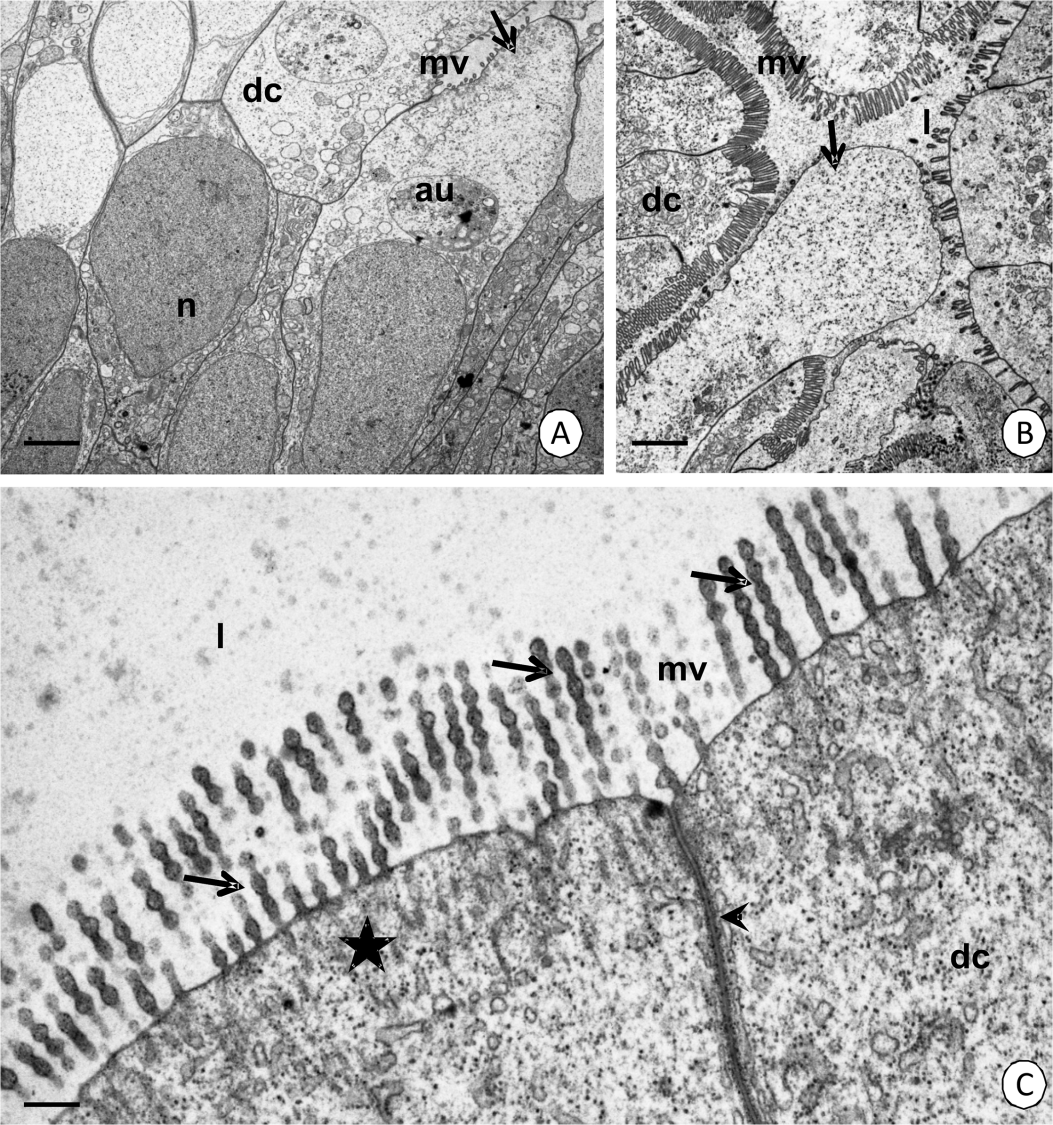
Secretion in the midgut epithelium. (A-B) Apocrine secretion in the intestine. Digestive cells (dc) with distinct evaginations of the apical membrane (arrows). (A) TEM. Bar = 1.7 µm. (B) TEM. Bar = 1.3 µm. (C) Microapocrine secretion: small bulges of microvilli (arrows) of the digestive cells (dc). TEM. Bar = 0.33 µm. Autophagosome (au), midgut lumen (l), microvilli (mv), nucleus (n), cortical layer of the apical cytoplasm (star), smooth septate junction (arrowhead).

The substances that are produced in the cytoplasm of the digestive cells are released into the midgut lumen due to the apocrine and microapocrine secretion. At the beginning of apocrine secretion, the apical membrane loses its microvilli and gradually forms an evagination that protrudes into the midgut lumen. Many organelles are shifted into the evagination, which gradually enlarges and eventually the evagination separates from the entire cell into the midgut lumen as a secretory bulge (Figs 1E, 6A and 6B). In the microapocrine secretion, small vesicles filled with an electron-lucent content protrude into the microvilli forming small bulges. When the bulges enter the apical surface of the microvilli, they are cut and discharged into the intestinal lumen (Fig 6C).

### The structure and ultrastructure of hepatopancreas

The hepatopancreas is a large and lobular organ that is composed of two large diverticles, which are secondarily divided into the blind-end tubules (Fig 1B and 1C). It is lined with the simple columnar epithelium, which rests on the non-cellular basal lamina and is separated from the body cavity by a circular layer of visceral muscles. Each tubule is composed of three distinct regions—distal, differentiation and proximal (Fig 1E).

#### Distal zone of hepatopancreatic tubules

**Fig 7:**
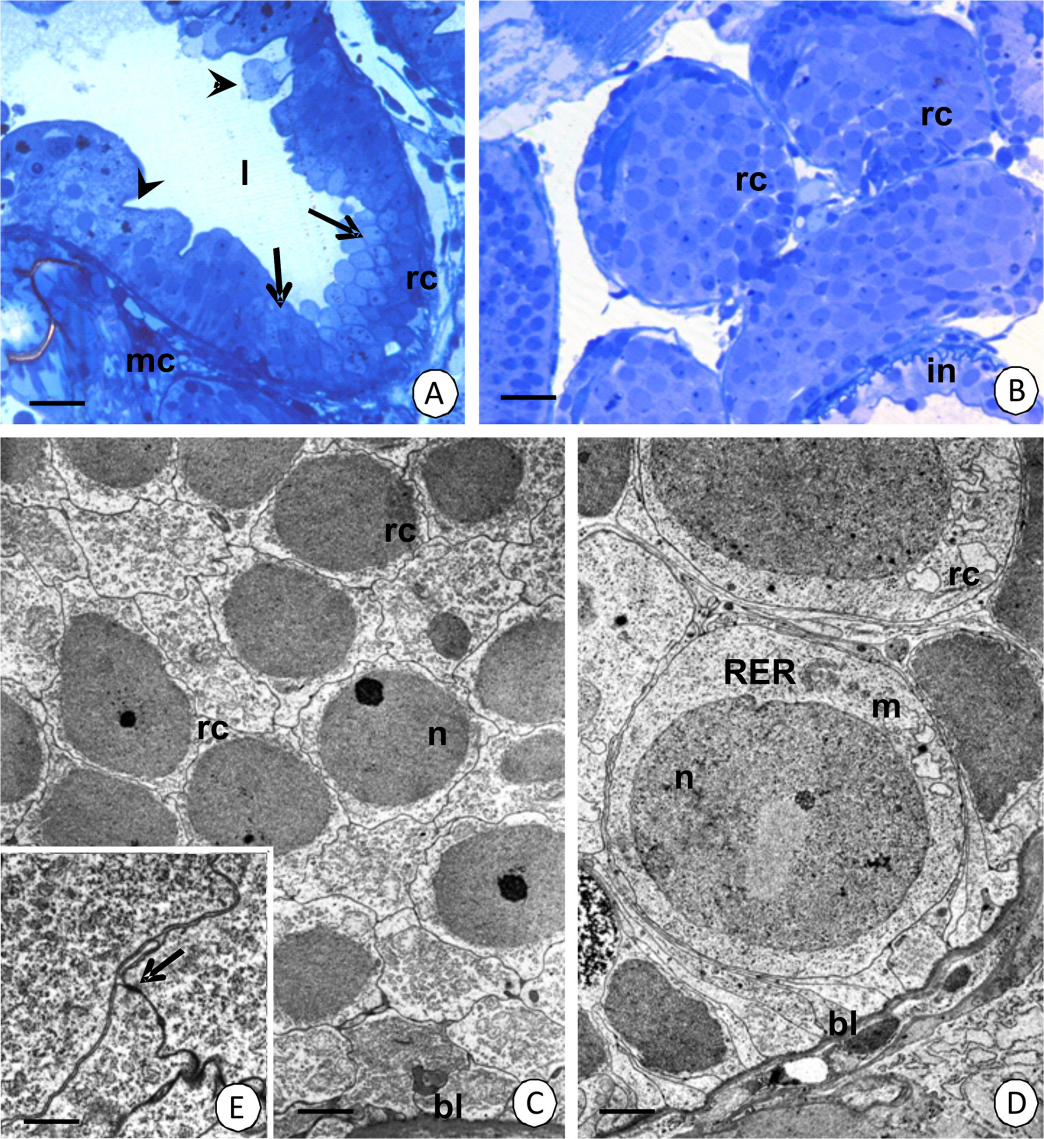
Distal region of hepatopancreatic tubules in *N*. *heteropoda*. (A) Longitudinal section through the distal (arrows) and differentiating (arrowheads) regions of tubule with regenerative cells (rc). Light microscopy. Bar = 26 µm. (B) Cross section through the distal region with regenerative cells (rc). Light microscopy. Bar = 19 µm. (C) Regenerative cells (rc). TEM. Bar = 3.15 µm. (D) Regenerative cells (rc). TEM. Bar = 2.43 µm. (E) Septate junctions (arrow) between regenerative cells. TEM. Bar = 1 µm. Hepatopancreatic lumen (l), visceral muscles (mc), intestine (in), basal lamina (bl), nucleus (n), mitochondria (m), cisterns of the rough endoplasmic reticulum (RER).

The distal region is formed by the regenerative cells = embryonic cells (Figs 1E, 7A and 7B). Their basal membrane does not form any folds and the cytoplasm is poor in organelles during the interphase (Fig 7C and 7D). Some mitochondria and cisterns of the rough endoplasmic reticulum are distributed around the oval-shaped nucleus which has small patches of the heterochromatin (Fig 7C and 7D). Between the regenerative cells septate junction as the intercellular junctions were observed (Fig 7E). The mitotic divisions of the regenerative cells were confirmed by anti-BrdU labeling (Fig 5C) and anti-phosphohistone H3 labeling (Fig 5D).

#### Medial (differentiation) zone of hepatopancreatic tubules

**Fig 8:**
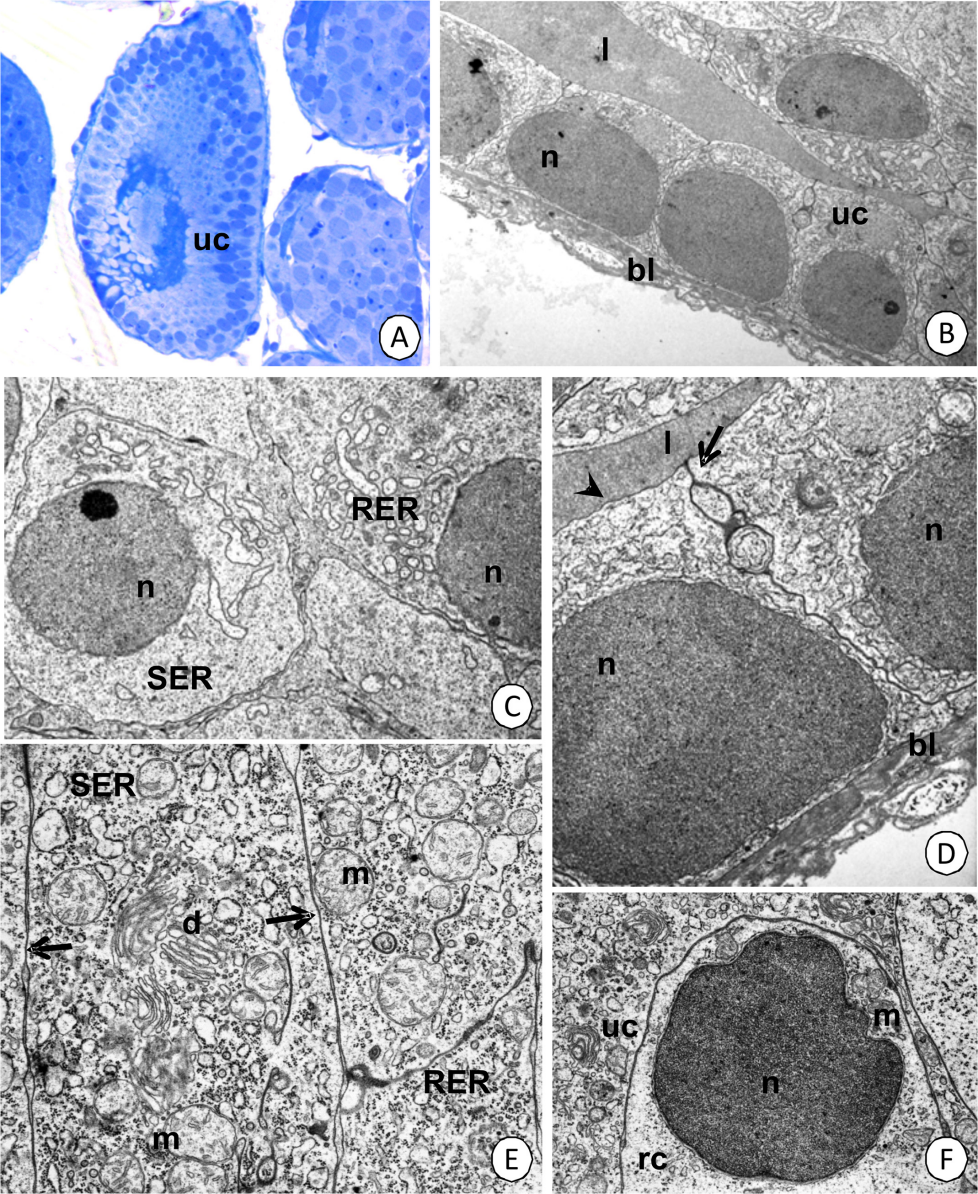
Differentiation region of hepatopancreatic tubules in *N*. *heteropoda*. (A) Cross section through the differentiation region. Light microscopy. Bar = 26 µm. (B) Longitudinal section through the differentiating region of tubule. TEM. Bar = 2.2 µm. (C) Cytoplasm of undifferentiated cells. TEM. Bar = 1.7 µm. (D) A higher magnification of (B). Apical membrane without any folds or microvilli (arrowhead) and distinct smooth septate junctions (arrows) between adjacent cells. TEM. Bar = 0.9 µm. (E) Cytoplasm rich in organelles. Gap junctions (arrows) between adjacent cells. TEM. Bar = 0.7 µm. (F) Sporadic regenerative cells (rc) with cytoplasm that was poor in organelles. TEM. Bar = 1 µm. Undifferentiated cells (uc), hepatopancreatic lumen (l), basal lamina (bl), nucleus (n), cisterns of the rough (RER) and smooth (SER) endoplasmic reticulum, mitochondria (m), Golgi complexes (d).

The medial region is formed by undifferentiated cells that differentiate (Figs 1E, 7A, 8A and 8D) into two types of cells, which are described below (see proximal region). Initially, all of the cells have a cubic shape and their cytoplasm is rich in mitochondria, Golgi complexes, cisterns of the rough and smooth endoplasmic reticulum (Fig 8C–8E). Their nuclei have an oval shape (Fig 8B–8D). The apical membrane is smooth and does not form any folds or microvilli. Smooth septate junctions and gap junctions can be distinguished between adjacent cells (Fig 8D); however, no septate junctions occurred. However, sporadic regenerative cells with the cytoplasm that was poor in organelles were observed (Fig 8F). No mitotic divisions of the regenerative cells were observed in this region.

#### Proximal zone of the hepatopancreatic tubules

**Fig 9:**
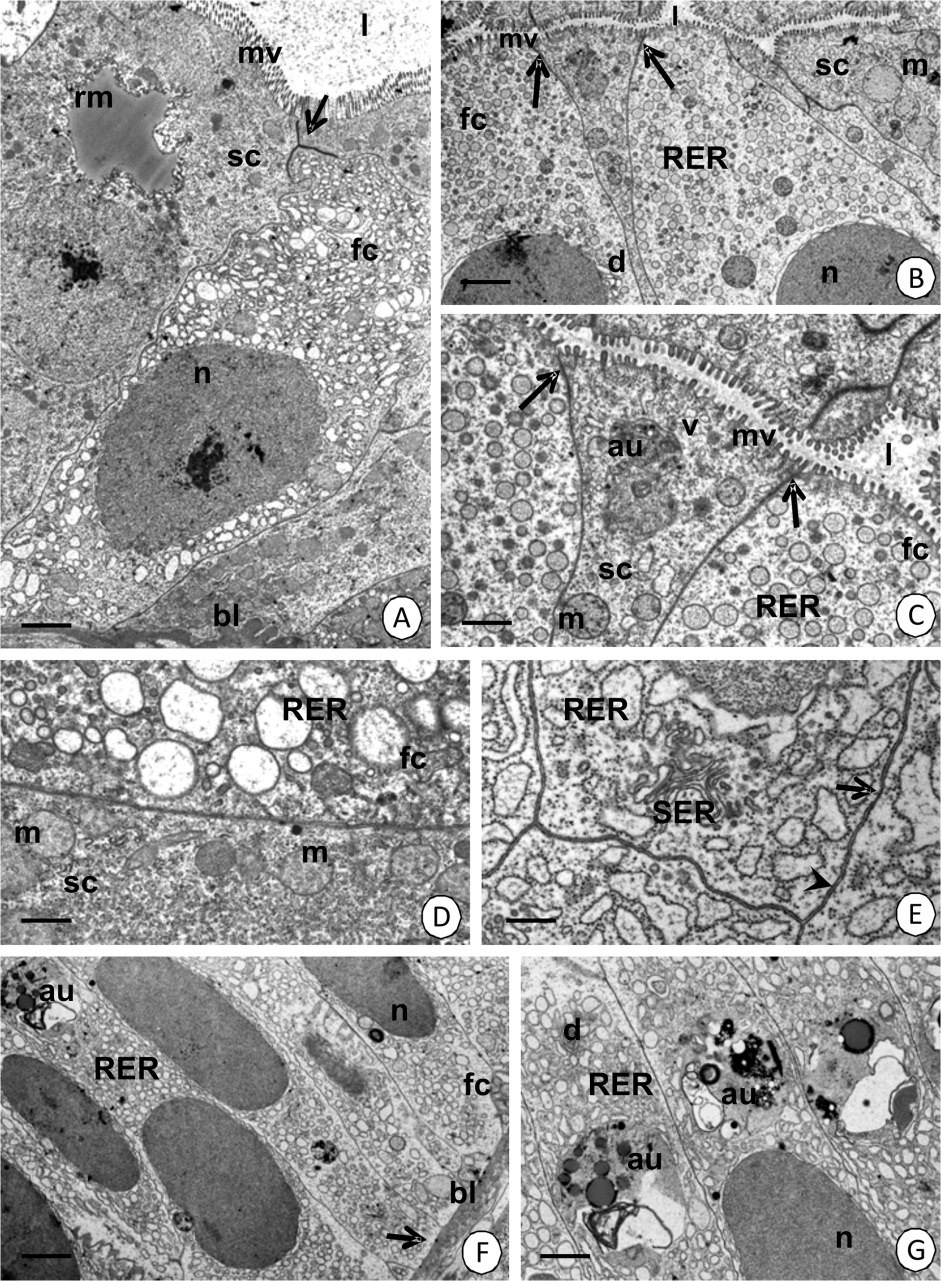
Proximal region of hepatopancreatic tubules in *N*. *heteropoda*. (A) Two types of cells were distinguished in the epithelium of the proximal region in hepatopancreatic tubule: type I—fibrillar cells (fc) and type II—storage cells (sc). TEM. Bar = 1.8 µm. (B) Fibrillar cells (fc) and storage cell (sc). TEM. Bar = 2.7 µm. (C) A higher magnification of (B). Fibrillar cells (fc)—a cortical layer with electron-lucent vesicles (v) and storage cell (sc). TEM. Bar = 1.5 µm. (D) The cytoplasm of fibrillar (fc) and storage (sc) cells. TEM. Bar = 0.7 µm. (E) Gap junctions (arrow) and septate junctions (arrowheads). TEM. Bar = 0.5 µm. (F) Fibrillar cells (fc). Basal membrane with small folds (arrows). TEM. Bar = 2.7 µm. (G) Autophagosomes (au) accumulated above the nucleus (n). TEM. Bar = 1.7 µm. Hepatopancreatic lumen (l), microvilli (mv), nucleus (n), basal lamina (bl), smooth septate junctions (arrows), reserve material (rm), cisterns of the rough (RER) and smooth (SER) endoplasmic reticulum, Golgi complexes (d), mitochondria (m), autophagosome (au).

Two types of cells can be distinguished in the epithelium of the proximal region in the hepatopancreatic tubule—**type I (fibrillar cells) and type II (storage cells)** (Figs 1E and 9A).

**The type I** cells (fibrillar cells) comprise about 70% of the epithelial cells. No regionalization in the distribution of organelles was observed. The apical membrane forms microvilli whose filaments enter the apical cytoplasm forming a thin cortical layer (Fig 9B and 9C). Only single small vesicles with an electron-lucent content and cisterns of the rough endoplasmic reticulum and free ribosomes are present in the cortical layer (Fig 9C). A nucleoplasm, which is of a medium electron-density, occupies the oval-shaped nucleus (Fig 9A, 9B and 9F). The entire cytoplasm is abundant in cisterns of the rough and smooth endoplasmic reticulum, Golgi complexes, but only single mitochondria were present (Fig 9A–9G). The well-developed rough endoplasmic reticulum was in the form of rounded vesicles of various sizes (Fig 9D and 9E). The basal membrane of these cells forms small folds (Fig 9A and 9F). There was no reserve material in the cytoplasm, which was PAS-negative (Fig 3D), Sudan Black B-negative (Fig 3E) and BPB-negative (Fig 3F).

**Fig 10:**
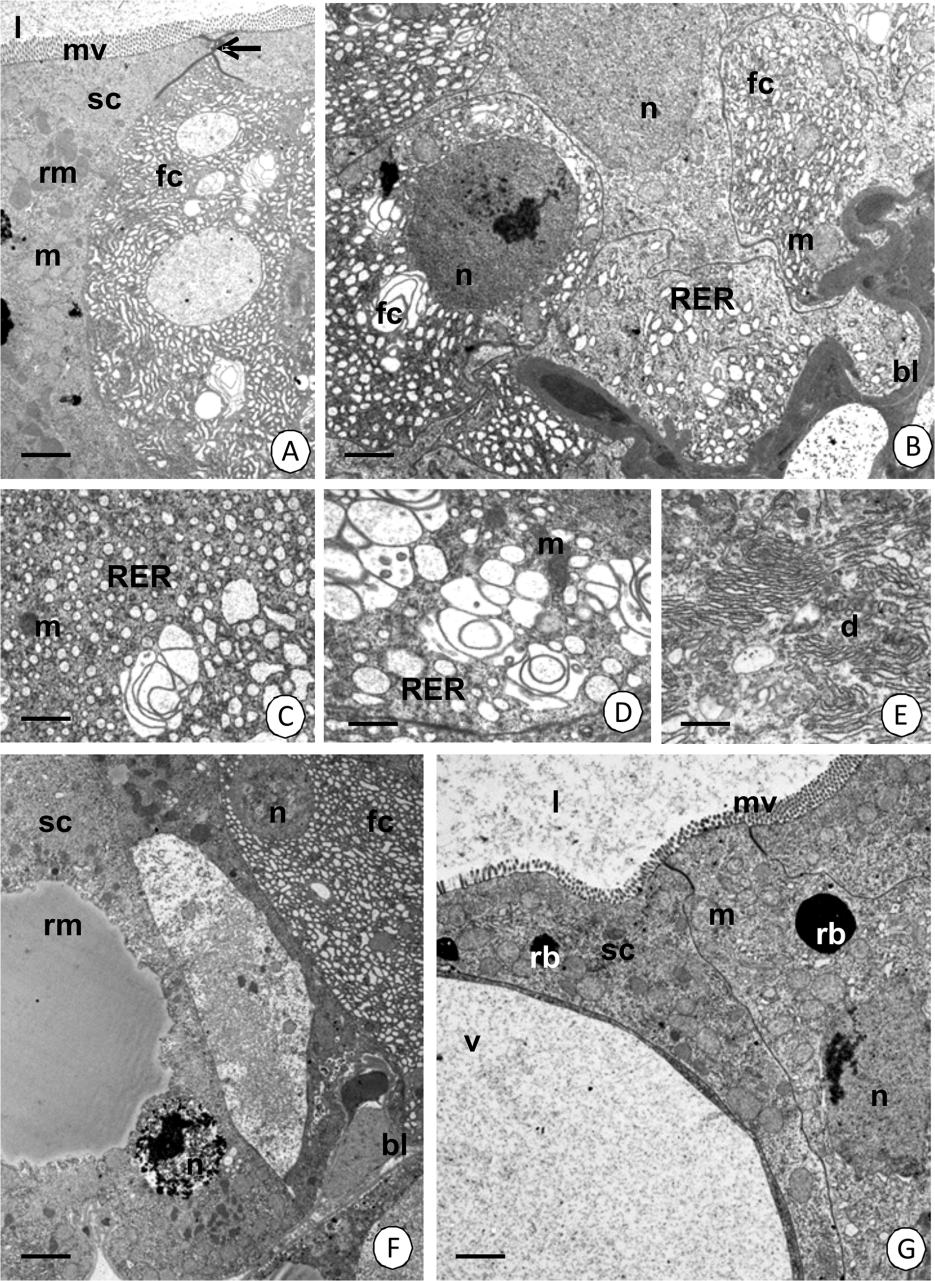
Changes in the fibrillar (fc) and storage (sc) cells according to their distance from the differentiation region of the tubule in *N*. *heteropoda*. TEM. (A) Bar = 1.7 µm. (B) Bar = 1.14 µm. (C) Bar = 1 µm. (D) Bar = 5.4 µm. (E) Bar = 6.5 µm. (F) Bar = 1.44 µm. (G) Bar = 1.32 µm. Hepatopancreatic lumen (l), microvilli (mv), basal lamina (bl), nucleus (n), reserve material (rm), vacuole (v), mitochondria (m), cisterns of the rough endoplasmic reticulum (RER), Golgi complexes (d), residual bodies (rb).

However, it was observed that the type I cells in each tubule gradually change their ultrastructure along the length of the epithelium. If the distance from the differentiation region of the tubule is longer, the cytoplasm begins to become electron-dense. Simultaneously, autophagosomes appear above the nucleus (Fig 9F and 9G). The number of cisterns of the rough and smooth endoplasmic reticulum and Golgi complexes gradually increases together with an enlargement of their dimensions (Fig 10A–10D) and the number of mitochondria decreases.

**Type II** cells (storage cells) comprise about 30% of the epithelial cells. No regionalization in the distribution of organelles was observed. The apical cell membrane forms microvilli (Fig 9A–9C), but no distinct cortical layer was distinguished. The entire cytoplasm is poor in organelles such as cisterns of the rough and smooth endoplasmic reticulum, but it is rich in mitochondria and free ribosomes (Fig 9C and 9D). Autophagosomes appear in the apical cytoplasm (Fig 9C). Additionally, the reserve material (Fig 9A), which is PAS-positive (Fig 3D), Sudan Black B-positive (Fig 3E), but BPB-negative, accumulates (Fig 3F). Type II cells, like type I cell, gradually change their ultrastructure along the epithelium. If the distance from the differentiation and distal zones of the tubule is longer, the amount of the reserve material (Fig 10A and 10F) and cisterns of the rough endoplasmic reticulum (Fig 10E) increases significantly. Simultaneously, large vacuoles that have an electron-lucent content (Fig 10F and 10G) and residual bodies such as the remains of the autophagy (Fig 10G) appear. The content of these vacuoles can be released into the hepatopancreatic lumen due to apocrine secretion.

Smooth septate junctions (in apical regions) (Figs 9A–9C, 10A), gap junctions and septate junctions (below the smooth septate junctions up to the basal cell membrane) (Fig 9E) occur between the adjacent cells that form the epithelium of hepatopancreas in its proximal region.

## Discussion

### Structure and ultrastructure of the midgut

The endodermal region (midgut) of the digestive system of Crustacea forms a tube-shaped organ that is devoid of any diverticula, has anterior caeca, is differentiated into the midgut (intestine) and large midgut gland, or the midgut can be replaced by a large hepatopancreas [2–6,8,18–21]. The tube-shaped midgut spreads along the entire length of the body cavity, while the hepatopancreas usually arises ventrally from the anterior margin of the midgut. The hepatopancreas, which occupies much of the cephalothoracic cavity, is composed of two pairs of blind-ending diverticles or lobes, which are secondarily divided into numerous blind-ended tubules. However, the number of hepatopancreatic diverticles or lobes can vary among different crustacean species [1]. Additionally, the hepatopancreatic tubules are subdivided into three distinct zones—distal, medial and proximal, relative to their distance from the intestine [22,23]. The midgut of *N*. *heteropoda* shares similarities with the midguts of other species that belong to Malacostraca. It is composed of a tube-shaped intestine and a large lobular hepatopancreas whose tubules are subdivided into distal, medial and proximal zones. The midgut is lined with an epithelium that rests on the non-cellular basal lamina. The epithelium of Crustacea consists of different cell types—R-cells (resorptive), B-cells (blister-like), M-cells (midgut, basal), F-cells (fibrillar) and E-cells (embryonic). In some crustaceans, only two types of cells have been detected in endodermal epithelium—B and S-cells [21,24]. Each cell type has a specific role in digestion (Table 1) [3,12,23,25]. In *N*. *heteropoda* we have described different types of cells—digestive and regenerative cells in the intestine and fibrillar, storage and regenerative cells in the hepatopancreatic tubules.

**Table 1:** The role and the localization of epithelial cells in endodermal region of the digestive system in Crustacea.

Types of cells	Functions	References	Localization	References
R-cells	detoxifying and recycling by accumulation of lipids, polysaccharides and metals, supply the energy during periods of starvation, molting and reproduction	[10,22,26]	Hepatopancreas: proximal zone	[6,12,28]
			Hepatopancreas: proximal and medial zones	[23]
			Hepatopancreas: medial zone	[7,31,32]
			Hepatopancreas: throughout the length of the organ	[29,33]
B-cells	intracellular digestion, accumulation of the absorbed materials and secretion	[27,28], **our studies**	Hepatopancreas: medial and distal zones	[23]
			Hepatopancreas: medial zone	[31]
			Hepatopancreas: proximal zone	[28,29,31], **our studies**
M-cells	lipids and polysaccharides accumulation	[13]	Hepatopancreas: throughout the length of the organ	[30]
			Absent	[30]
F-cells	synthesis and secretion of digestive enzymes	[3,22,28–30], **our studies**	Hepatopancreas: medial and proximal zones	[29,30,31]
			Hepatopancreas: throughout the length of the organ	[23]
			Hepatopancreas: proximal zone	**our studies**
E-cells	midgut regeneration	[6,14,20,22, 31], **our studies**	Intestine:anterior region	**our studies,** [22]
			Hepatopancreas: distal and medial zone	**our studies,** [34]
			Absent	[4,15,16,19]
D-cells	synthesis and secretion of digestive enzymes	**our studies**	Intestine	**our studies**

The digestive cells of the intestine in *N*. *heteropoda* have an ultrastructure that is characteristic of invertebrate cells that take part in the synthesis and secretion of enzymes, and that have a well-developed endoplasmic reticulum [4,35–37]. Additionally, two types of secretions occur in the digestive cells—apocrine and microapocrine. These cells do not participate in the accumulation of the reserve material. As to their functions, the digestive cells of the intestine in *N*. *heteropoda* correspond to F-cells [23]. However, the fibrillar cells in the hepatopancreatic tubules have numerous cisterns of the endoplasmic reticulum, which gives them a fibrillar appearance, which was not observed in digestive cells of the species examined. However, the data connected with the ultrastructure of the digestive cells of the intestine in crustaceans are rather general [3,4], while this paper describes their precise ultrastructure. In conclusion, the digestive cells described in *N*. *heteropoda* are similar to the digestive cells that have been described for other arthropods, e.g., insects, myriapods [4,35–41]. Therefore, we called them D-cells (digestive cells) (Table 1).

There are still controversies connected with all of the types of cells which form the epithelium of hepatopancreas in Crustacea. Their appearance in different zones of hepatopancreatic tubules has been presented in Table 1. In the case of *N*. *heteropoda*, we have described only three types of cells, which we called fibrillar cells (type I), storage cells (type II) and regenerative cells (E-cells). Their ultrastructure suggests that fibrillar cells correspond to F-cells, while storage cells are related to B-cells as they are responsible for the storage of the reserve material [22]. F- and B-cells are present only in the proximal zone of the hepatopancreatic tubules. The distal zone has only E-cells (embryonic), while the medial zone represents a differentiated region in which all cells have the same structure, but they begin to differentiate into F- and B-cells. However, if the distance from the medial and distal zones of the hepatopancreatic lumen is longer, numerous alterations in the cytoplasm of F- and B-cells occur. Large vacuoles appear in the cytoplasm of the B-cell in *N*. *heteropoda*, thus giving this cell the appearance of a B-cell as has been described in many crustaceans [22]. However, in the literature, we can also find controversies on the origin of the types of cells described here. Sousa and Petriella [12] stated that F-, R- and B-cells originated independently from E-cells. Correira et al. [7] described the differentiation of E-cells into distinct R- and F-cells, while B-cells appear in the proximal zone as cells that have a large vacuole inside. Several studies do not present the differences between these two types of cells [10,42]. Our studies have shown that in *N*. *heteropoda* distinct regionalization in cell activity occurs along the length of the hepatopancreatic tubules. We distinguished a distal zone with E-cells, a medial zone with some E-cells and differentiating cells and eventually, a proximal zone that has two types of cells—F and B-cells. Additionally, cells that are at different stages of their activity were found running along the whole length of the proximal zone. If the cell F or B is closer to the beginning of the hepatopancreatic tubule, it is more active (e.g., secretion is observed only in this region).

### E-cells as midgut regenerative cells

Embryonic cells (E-cells) occur among the cell types in the midgut epithelium of crustaceans that were described above. E-cells, being able to proliferate and differentiate, correspond to arthropods midgut regenerative cells = midgut stem cells [6,14,20,22,31, 35–41]. They have been described as small basal cells that are scattered between the other epithelial cells [1,3,12,31,43]. Their divisions have been observed in crustaceans in the distal and medial zones, while no mitosis was observed in the proximal zone of the hepatopancreatic tubules [22]. However, in some cases mitotic divisions of E-cells were not confirmed [3] or these cells have not been observed in the midgut epithelium [4,15,16,19]. It has been suggested that a short life span causes epithelial degeneration to be rare and therefore the midgut epithelium can survive without regeneration. Additionally, another mechanism, which enables the proper functioning of the midgut epithelium occurs—the process of autophagy [4]. Regenerative cells (E-cells) in *N*. *heteropoda* are present only in the anterior region of the intestine and in the distal and medial zone of the hepatopancreatic tubules. Their mitotic divisions have been confirmed only in the intestine and the distal zone. According to the literature, we have concluded that they play the role of the midgut regenerative cells that are responsible for epithelial regeneration [35–41,44,45]. E-cells replace damaged cells of the intestine epithelium and form the distal zone of the hepatopancreatic tubules. Therefore, they should differentiate in all types of hepatopancreatic epithelium [3,12,23,28,29,31,46]. The differentiation of E-cells has not been observed in the intestine of *N*. *heteropoda*, which may be connected with, e.g., molting, circadian rhythms or reproduction as has been described for many arthropods [35,37,38,41,44,45]. However, this process was detected in the medial zone of hepatopancreatic epithelium where first they have a cubic shape and gradually numerous organelles accumulate in their cytoplasm in the freshwater shrimp that was examined in this study. While moving into the proximal zone, they achieve the character of F- or B-cells. Therefore, we can conclude that E-cells play the role of midgut regenerative cells = midgut stem cells in Crustacea.

Our studies have also revealed the presence of intercellular junctions between adjacent E-cells acting as midgut regenerative cells. Septate junctions were detected between E-cells and D-cells in the intestine, between E-cells in the distal zone of hepatopancreatic zone and between E-cells and differentiating cells of the medial zone. Septate junctions are involved in adherens of neighboring cells and they can also form a partial permeability barrier [39,40,47]. The existence of intercellular junctions between midgut stem cells or midgut stem cells and midgut epithelial cells in invertebrates has not been studied precisely. Therefore, this is the first report on the existence of intercellular junctions that are connected with the E-cells of Crustacea.

## Conclusions

During our studies we found that: (a) the endodermal region of the digestive system is composed of an intestine and a hepatopancreas; (b) each tubule of the hepatopancreas shows distinct regionalization with three zones—distal, medial and proximal; (c) two types of cells form the intestine epithelium—D- and E-cells; (d) three types of cells form the hepatopancreatic tubules—E-, F- and B-cells; (e) E-cells play the role of regenerative cells; (f) distinct intercellular junctions appear between E-cells; (g) B- and F-cells are present at different stages of their activity.

## Material and Methods

### Material

The studies were performed on adult males and females of the freshwater shrimp *Neocaridina heteropoda* (Crustacea, Malacostraca, Decapoda) (Fig 1A). The individuals for the studies were obtained from local shrimp breeders and kept in 30-liter aquaria in the laboratory (no specific permissions were required for breeding). The environmental conditions in the aquaria were strictly controlled, i.e. temperature of 24°C, pH of 7 and total water hardness of 15^0^d. The *N*. *heteropoda* specimens were fed with food that is produced for freshwater shrimps, which consists of mainly vegetable by-products, algae and fish by-products. The principles of laboratory animal care were followed, as well as specific national laws where applicable.

### Methods

#### X-ray computed tomography

X-ray Microtomography (XMT) is a non-destructive, computed-aid visualization technique that employs an electron X-ray conical beam to visualize the internal structures of tissues and organs. It allows high-resolution morphological and anatomical data to be created in three dimensions in a very fast and non-invasive way without having to cut the material.

The adult specimens of freshwater shrimp were fixed with 2% trichloromethane (2 minutes, 24⁰C). The samples were scanned using an XMT v|tome|x s scanner (GE Sensing & Inspection Technologies, phoenix|x-ray, Wunstorf, Germany), which is equipped with two X-ray sources—a 240V/320W microfocus and a 180V/15W nanofocus. The temperature-stabilized 16'' DXF detector provides superior contrast. All of the scans were performed using the nanofocus tube and X-ray power lower than the voxel size of the data matrix. The scanning parameters used during the tests are presented in Table 2. After scanning, the material was reconstructed using compatible phoenix datos|x 2.0 software. Drishti 2.4 was used to analyze the image of the adult form.

**Table 2:** The scanning parameters used during the tests using X-ray microtomography.

**Voltage (kV)**	40
**Current (µA)**	290
**Power (W)**	11,6
**Filter (mm)**	0,1 Cu
**Number of projections**	2000
**Resolution (µm)**	11,663
**Timming (ms)**	131
**Average**	3
**Skip**	1
**Scan time (min)**	27
**Reconstructed Volume size (voxels)**	2024x2024x 396

#### Light and transmission electron microscopy

Adult specimens of *N*. *heteropoda* were decapitated (15 females and 15 males) and prepared for the analysis and fixed with 2.5% glutaraldehyde in a 0.1 M sodium phosphate buffer (pH 7.4, 4°C, 2h), postfixed in 2% osmium tetroxide in a 0.1 M phosphate buffer (4°C, 1.5 h), dehydrated in a graded series of concentrations of ethanol (50, 70, 90, 95 and 4x100%, each for 15 min) and acetone (15 min) and embedded in epoxy resin (Epoxy Embedding Medium Kit; Sigma). Semi- and ultra-thin sections were cut on a Leica Ultracut UCT25 ultramicrotome. Semi-thin sections (0.8 µm thick) were stained with 1% methylene blue in 0.5% borax and observed using an Olympus BX60 light microscope. Semi-thin sections that were not stained with the 1% methylene blue were used for the histochemical analyses (see below). Ultra-thin sections (70 nm) were stained with uranyl acetate and lead citrate and examined using a Hitachi H500 transmission electron microscope.

#### Detection of glycogen and polysaccharides (PAS method)

Semi-thin sections were treated with a 2% solution of periodic acid (10 min, room temperature) and stained with Schiff’s reagent (24 h, 37°C). After washing the sections with water, the slides were analyzed using an Olympus BX60 light microscope.

#### Detection of proteins

Semi-thin sections were treated with a 1% solution of periodic acid (10 min, room temperature) and stained with bromophenol blue (BPB) (24 h, 37°C). After washing the sections with water, the material was examined using an Olympus BX60 light microscope.

#### Detection of lipids

Semi-thin sections were stained with Sudan black B at room temperature (20 min). After washing the sections with ethanol and water, the slides were examined using an Olympus BX60 light microscope.

#### Fluorescence microscopy—labeling of nuclei and filamentous actin

After decapitation five females and five males of *N*. *heteropoda* were embedded without fixation in a tissue-freezing medium (Jung) and rapidly frozen. Cryostat sections (5 μm thick) were placed on 1% gelatin-coated slides. All of the subsequent steps were carried out at room temperature. Initially, the slides were washed in Tris-buffered saline (TBS; 5 min) and in 0.1% Triton X-100 in TBS (5 min). Then the material was stained with rhodamine-phalloidin (40 min in darkness). After washing in TBS (five times, 10 min each), the material was labeled with Hoechst 33342 (1µg/ml, 10 min in darkness). The slides were analyzed using an Olympus BX60 fluorescence microscope.

#### Fluorescence microscopy—BrdU labeling of proliferating cells

Proliferating cells were identified by labeling with 5-bromo-2’-deoxyuridine-5’-monophosphate (BrdU). Two males and two females of the species that were examined were injected with 50 mg BrdU/kg body weight (BrdU dissolved in TBS). After 1 h the animals were killed and decapitated. The material was embedded in a tissue-freezing medium (Jung) without fixation. Cryostat sections (5 μm thick) were mounted on 1% gelatin-coated slides. After washing in phosphate-buffered saline (PBS; 3 min, room temperature) and incubation in 2N HCl for 60 minutes (37°C), the material was incubated for 1 h (in darkness, room temperature) with 50 μg/ml anti-BrdU antibodies conjugated to fluorescein (Roche) diluted in PBS with 0.1% BSA, then washed in TBS (2x, 5 min, room temperature). Finally, the material was stained with Hoechst 33342 (1µg/ml) for 10 minutes at room temperature. The slides were analyzed using an Olympus BX60 fluorescence microscope.

#### Fluorescence microscopy—Immunolabeling with anti-phosphohistone H3 antibody

The slides that had been prepared for the ***Labeling of nuclei and filamentous actin*** were also used for immunolabeling with anti-phosphohistone H3 antibody. All of the subsequent steps were carried out at room temperature. After washing the slides in TBS (5 min) and 0.1% Triton X-100 in TBS (5 min), the sections were covered with 1% BSA in TBS (30 min). Then the material was incubated overnight in a 1:100 dilution of anti-phosphohistone H3 antibodies (Millipore) in 1% BSA in TBS. After washing the slides with TBS (2x, 5 min), they were incubated for 1 h (in darkness) in a 1:200 dilution of goat anti-rabbit IgG Alexa-Fluor 488 conjugated secondary antibodies in 1% BSA in TBS. Then the material was stained with Hoechst 33342 (1µg/ml, 10 min) and mounted in 50% glycerol. The sections were analyzed using an Olympus BX60 fluorescence microscope.

## Supporting Information

S1 Video 3D representation of the midgut in *N*. *heteropoda*. Intestine (red), hepatopancreas (blue), stomach (green) as the fragment of foregut which adheres intestine.
(AVI)

S1 Author Summary (DOC)

S1 Abstract ICIM (TIF)
