# Supplementary material for: Structure and Ultrastructure of the Endodermal Region of the Alimentary Tract in the Freshwater Shrimp Neocaridina heteropoda (Crustacea, Malacostraca)
Source: PLoS One. 2015 May 21;10(5):e0126900. doi: 10.1371/journal.pone.0126900 (PMC4440751; doi:10.1371/journal.pone.0126900)
Supplement: S1 Author Summary — (DOC) [file pone.0126900.s002.doc]

**Author Summary**

The digestive system of invertebrates is composed of ectodermal fore- and hindguts, while its Middle endodermal region is called as the midgut. This part can consist of an intestine and a hepatopancreas. As the aim of our study we chose freshwater shrimp which is widely bred all over the world, so its structure and habitats are of interests of many breeders. In our studies we used X-ray microtomography to show the organization of the endodermal region of the digestive system in 3D. TEM, light and fluorescence microscopes let us to reveal the ultrastructure of all types of epithelial cells, which occurred in the intestine: D – and E- cells, and hepatopancreas: F, B and E-cells. We paid our attention on the functioning of the E – cells which are treated as the midgut stem cells. Therefore, their proliferation has been shown. Additionally, the description of distinct regionalization in hepatopancreatic tubules confirmed the statement that E – cells can also differentiate into the midgut cells. Here we also present the first report on the existence of an intercellular junction that is connected with the E-cells of Crustacea.

**Blurb**

1. X-ray microtomography reveals organization of the midgut in *Neocaridina heteropoda.*
2. Ultrastructure of epithelial cells in intestine and hepatopancreas with the emphasis on midgut stem cells has been shown.
