# Supplementary figures and images for: Structure and Ultrastructure of the Endodermal Region of the Alimentary Tract in the Freshwater Shrimp Neocaridina heteropoda (Crustacea, Malacostraca)
Source: PLoS One. 2015 May 21;10(5):e0126900. doi: 10.1371/journal.pone.0126900 (PMC4440751; doi:10.1371/journal.pone.0126900)

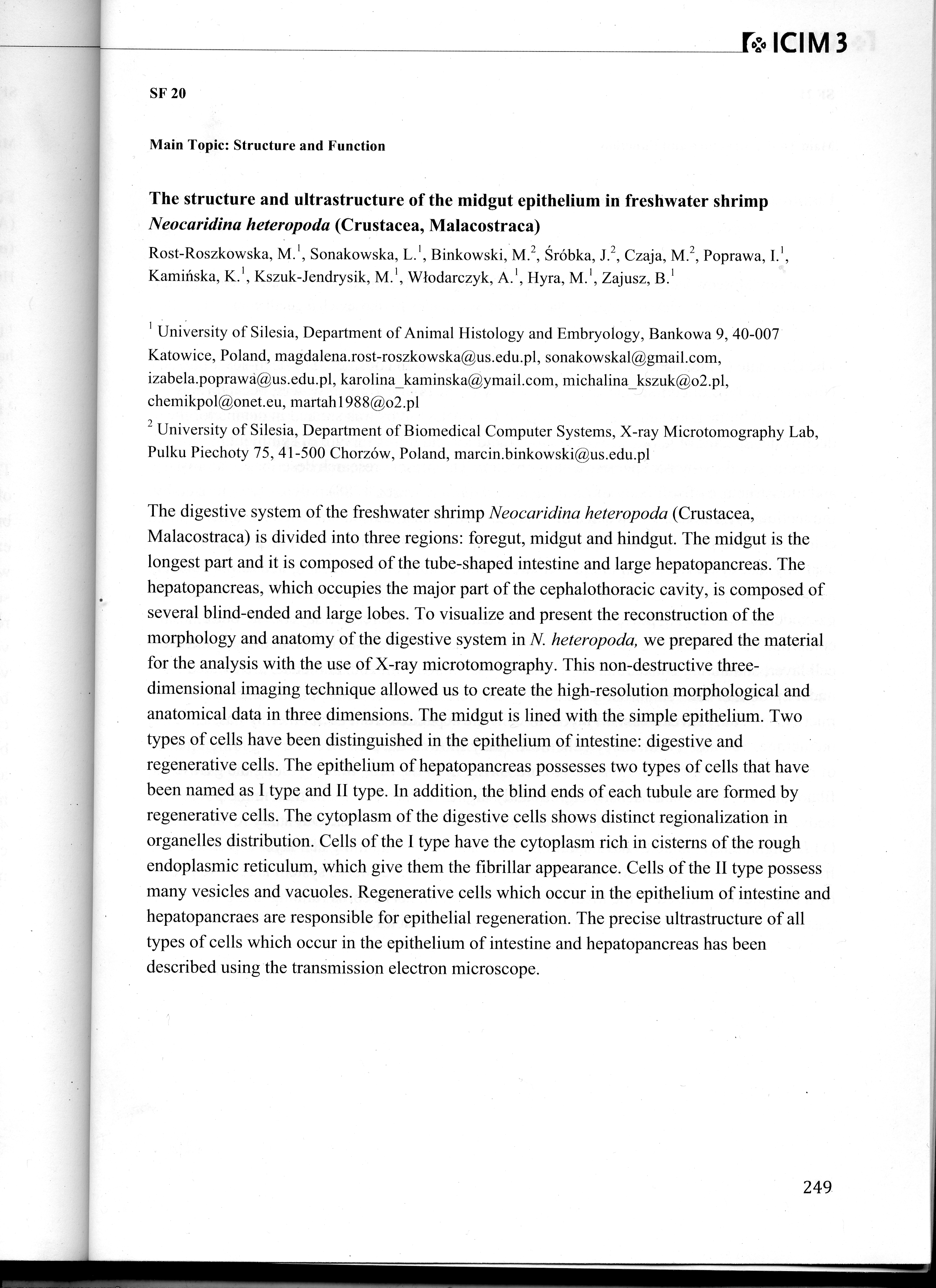

Supplement: S1 Abstract ICIM — (TIF) [file pone.0126900.s003.tif]
